# Conditional activation of an anti-IgM antibody-drug conjugate for precise B cell lymphoma targeting

**DOI:** 10.3389/fimmu.2023.1258700

**Published:** 2023-09-28

**Authors:** Katrin Schoenfeld, Julia Harwardt, Jan Habermann, Adrian Elter, Harald Kolmar

**Affiliations:** ^1^ Institute for Organic Chemistry and Biochemistry, Technical University of Darmstadt, Darmstadt, Germany; ^2^ Centre for Synthetic Biology, Technical University of Darmstadt, Darmstadt, Germany

**Keywords:** B cell receptor, antibody-drug conjugate, masked antibody, conditional activated antibody, MMP-9, matriptase, B cell lymphoma

## Abstract

Cancerous B cells are almost indistinguishable from their non-malignant counterparts regarding their surface antigen expression. Accordingly, the challenge to be faced consists in elimination of the malignant B cell population while maintaining a functional adaptive immune system. Here, we present an IgM-specific antibody-drug conjugate masked by fusion of the epitope-bearing IgM constant domain. Antibody masking impaired interaction with soluble pentameric as well as cell surface-expressed IgM molecules rendering the antibody cytotoxically inactive. Binding capacity of the anti-IgM antibody drug conjugate was restored upon conditional protease-mediated demasking which consequently enabled target-dependent antibody internalization and subsequent induction of apoptosis in malignant B cells. This easily adaptable approach potentially provides a novel mechanism of clonal B cell lymphoma eradication to the arsenal available for non-Hodgkin's lymphoma treatment.

## Introduction

Immunotherapies represent a broad and rapidly growing type of therapies having a substantial impact on cancer outcomes. Monoclonal antibodies (mAbs) are among the first groups of immunotherapies approved for anti-tumor treatment and are still of exceptional relevance in current treatment regimens (1). Rituximab, the first US Food and Drug Administration (FDA)-approved mAb implemented in oncology, has widely been administered in patients suffering from B cell non-Hodgkin's lymphoma (NHL). NHL is a heterogeneous group of neoplasms and the most frequently diagnosed adult hematological cancer, accounting for the seventh most common cancer and the ninth leading cause of cancer deaths in the US (2). Targeting the pan-B cell antigen CD20, rituximab exerts anti-tumor activity in four main ways, three of which rely on recruiting effector mechanisms from the patient's immune system such as complement-dependent cytotoxicity (CDC), antibody-dependent cell-mediated cytotoxicity (ADCC) and antibody-dependent phagocytosis (ADP) (3). A fourth proposed mechanism of action is the induction of apoptosis through both caspase-dependent and -independent mechanisms (3, 4). Although rituximab in combination with chemotherapy has tremendously improved the chance of cure for NHL patients, the clinical effectiveness of rituximab is ultimately limited by the development of treatment resistance. Notably, only 40% of the patients initially responding to rituximab have the ability to respond again after relapse (5, 6).

The B cell receptor (BCR) complex plays a pivotal role in the adaptive immune response. Comprising a membrane-bound immunoglobulin (Ig) and a non-covalently linked heterodimer composed of Igα and Igβ it is expressed on the surface of B lymphocytes with each B cell clone possessing a unique BCR of Ig isotype IgA, IgD, IgE, IgG, or IgM (7, 8). Previous reports have demonstrated that malignant B cells frequently express IgM BCRs (9–12). A subtype of the diffuse large B cell lymphoma (DLBCL) is activated B cell-like DLBCL, where it has been reported that IgM-positivity of tumor correlates with a poor prognosis and a shorter overall survival for patients (10–12). Harnessing the fact that clonal B cell cancers in most cases express BCRs of one Ig isotype, it might be possible to selectively deplete malignant B cells of the IgM isotype while sparing the majority of B lymphocytes expressing other isotype or no BCRs. However, therapeutic antibodies directed against IgM may not fully function in the body due to the presence of soluble IgM molecules in large amounts. In order to address the problem of selectivity and potential target-mediated drug disposition, an IgMxHLA-DR bispecific antibody targeting two B cell antigens has recently been engineered which demonstrated significant *in vitro* anti-tumor activity as well as efficacy and tolerability in non-human primate studies (13).

Besides improving specificity via multispecific cancer targeting, masking strategies have been developed allowing for conditional activation of antibodies in tumor tissue (14–16). The approach requires the generation of a suitable masking unit which prevents antibody-antigen interaction either by steric hindrance, e.g. by fusion of a bulky mask, or by specific binding to the antibody paratope, such as an epitope-mimetic or anti-idiotypic antibody fragment (14, 16). Antibody activation through demasking is typically mediated by proteases, such as serine proteases (e.g. matriptase), matrix metalloproteinases (e.g. MMP-2/MMP-9) and cysteine proteases (e.g. cathepsin S) frequently overexpressed in tumor tissues (17–19). Previous masking attempts put forth antibody therapeutics with improved safety profiles, while retaining anti-disease activity (20–24). The versatile probody therapeutic technology platform developed by CytomX Therapeutics has been applied to target a variety of receptors including CTLA-4, EGFR, as well as molecules considered undruggable because of their broad tissue expression, such as CD71 and EpCAM (25–27). The conditionally activated probody-drug conjugate CX-2029 (anti-CD71) demonstrated tumor regression and was well tolerated in patients with advanced solid tumors (28).

To combat resistance of current mAb-based therapies and improve the potency of biomolecules, antibody-drug conjugates (ADC) feature ideal properties for precise and efficient tumor targeting (29, 30). The first-in-class ADC to be FDA-approved for therapy was gemtuzumab ozogamicin (Mylotarg), in 2000 for the treatment of CD33-positive acute myeloid leukemia (AML) (31). Since then, 14 ADCs received worldwide market approval, besides over 100 ADC candidates being investigated in clinical stages at present (32). ADCs are typically composed of mAbs covalently bound to potent cytotoxic payload through synthetic (cleavable) linkers. However, there is ongoing optimization of certain parameters, including mAb specificity, linker technology, drug potency as well as stoichiometry and placement of warheads (30, 32). The mechanism leading to ADC's anti-tumor effect includes binding of the ADC to its target antigen that triggers ADC internalization and intracellular release of the payload which eventually mediates cytotoxic effects. Hence, candidate ADCs must be carefully selected regarding numerous properties influencing safety and efficacy. Particularly, the antigen to be targeted by the ADC must fulfill certain characteristics such as overexpression on the surface of cancer cells with minimal expression in normal tissue and the potency to rapidly internalize upon ADC binding (32). Since B cell NHL is currently treated with either chemotherapy or immunotherapy or a combination of both, it is anticipated that ADCs can be rational for NHL control.

**Figure 1:**
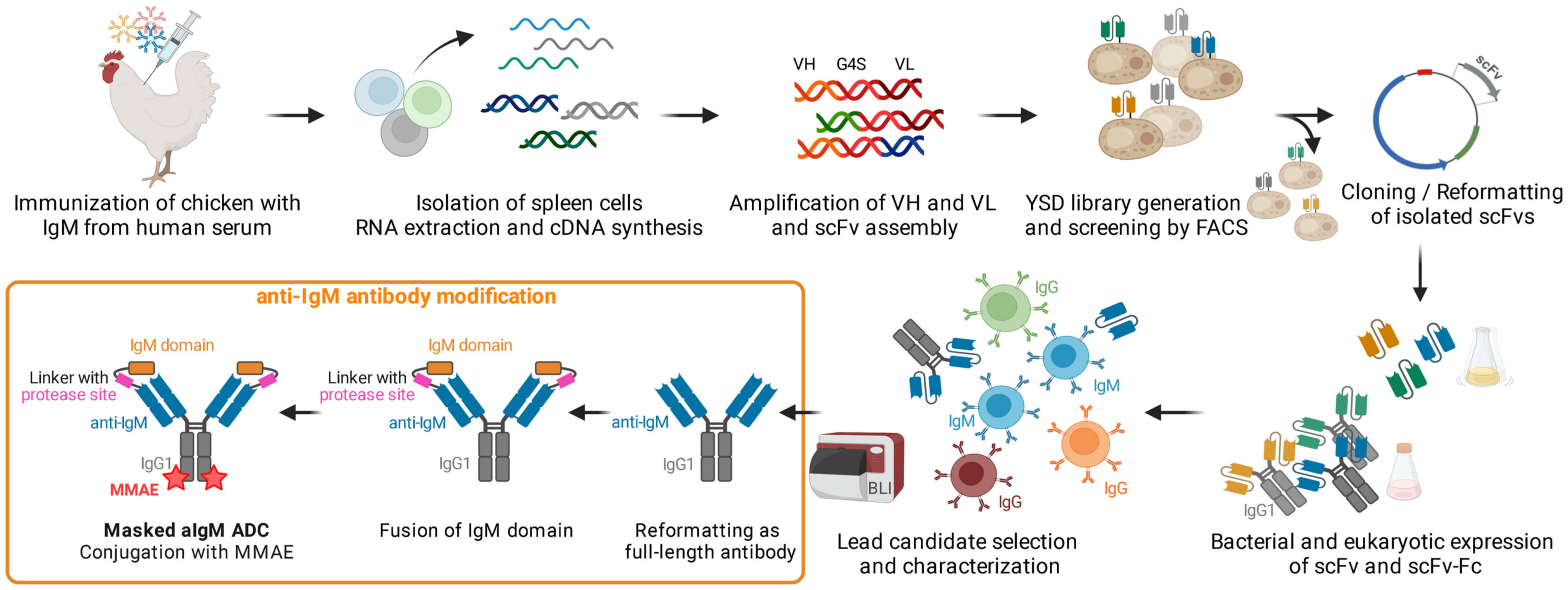
Concept Overview. Chicken immunization with IgM from human serum was followed by splenic RNA isolation and cDNA synthesis. Variable antibody domains were amplified and assembled as scFvs for yeast surface display and FACS. IgM binding scFvs were reformatted and cloned into bacterial and mammalian vectors for scFv/scFv-Fc expression. After selection and characterization of a lead candidate, the IgM binder was reformatted as full-length antibody, fused with the masking IgM domain and ultimately conjugated with MMAE resulting in a masked anti-IgM (aIgM) ADC. Created with BioRender.com.

In this study, we developed a proteolytically activatable IgM-directed antibody-drug conjugate for precise targeting of IgM-positive B cell lymphoma (**Figure 1**). Starting with the immunization of a chicken with IgM from human serum, we isolated IgM binders by single-chain variable fragment (scFv) immune library screening using yeast surface display (YSD) in combination with fluorescence-activated cell sorting (FACS). After expression and characterization of isolated binders in scFv format, full-length antibodies in Fab-Fc format were generated. With respect to potential off-target effects on healthy IgM-expressing B cells and capturing of antibodies by soluble IgM in the blood stream, we identified the antigenic constant Ig domain, derived from the IgM antigen, for antibody masking. The masking unit was genetically fused to the N-terminus of the anti-IgM light chain (LC) via a dual-protease cleavable linker addressable by matrix metalloproteinase-9 (MMP-9) and matriptase since these proteases are described to be overexpressed in B cell lymphoma (33, 34). The IgM-targeting antibody was further conjugated with the highly toxic and clinically proven chemotherapeutic agent monomethyl auristatin E (MMAE) imparting cytotoxic properties to the molecule (32). The resulting masked anti-IgM ADC demonstrated no significant interactions with different types of B cells. However, unmasking resulted in specific targeting and efficient killing of IgM-positive lymphoma cells while largely sparing other lymphocytes from chemotherapeutic damage.

## Results

### Design of protease-activated masked anti-IgM antibody-drug conjugates

**Figure 2:**
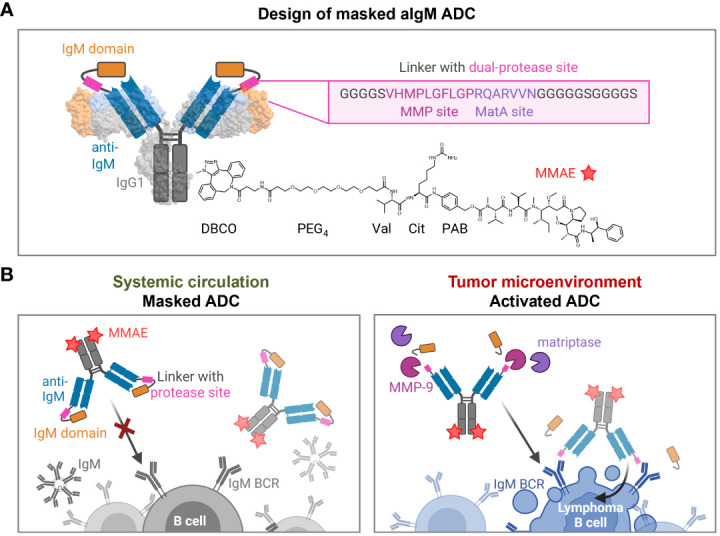
Design and mode of action of masked aIgM ADC. **(A)** Schematic representation of the masked aIgM ADC. The anti-IgM LC is (N-terminally) fused to an IgM domain via a linker with dual-protease site, the HC is (C-terminally) modified with DBCO-PEG_4_-Val-Cit-PAB-MMAE. DBCO, Dibenzocyclooctyne; PEG, polyethylene glycol; Val, valine, Cit: citrulline; PAB, p-aminobenzyl alcohol; MMAE, monomethyl auristatin E. Surface representation rendered with UCSF ChimeraX (35) from PDB: 1IGT/7XQ8. **(B)** Conceptional mode of action of the masked anti-IgM ADC. In systemic circulation the masked aIgM ADC is not able to bind to either soluble pentameric IgM nor membrane-bound IgM. Once reaching the tumor microenvironment, tumor-specific proteases such as MMP-9 or matriptase hydrolyze the linker connecting the aIgM antibody and the masking IgM domain. The activated aIgM ADC regains binding ability leading to specific ADC uptake and killing of IgM^+^ lymphoma B cells. Created with BioRender.com.

Based on a chicken-derived anti-IgM (aIgM) antibody, we designed an antibody-drug conjugate that is masked to overcome potential off-target effects towards circulating IgM^+^ B cells and interactions with soluble IgM in the blood stream (**Figure 2**). In our approach, the human IgM domain targeted by the antibody served as masking unit attached to the aIgM light chain. We assumed that the heavy chain CDRs are mainly responsible for antigen recognition as this was discovered in previous chicken-derived antibodies including common light chain approaches and is reinforced by the fact that chicken CDR3 of the VH tend to be longer and have much higher cysteine content leading to increased stability and complexity (36–39). Fusion of the masking unit was achieved via a synthetic linker (33 amino acids) comprising a dual-protease site (MMP site and MatA site) recognized by MMP-2/9 and matriptase (**Figure 2A**). Linker sequence and applicability to protease-activated antibodies in tumor context have been recently described by *Geiger et al.*, demonstrating a synergistic effect for the combination of the cleavage sites for MMP-2/9-matriptase compared to MatA site or MMP site linkers alone (21). For the generation of an ADC the mAb component was further provided with MMAE, since NHL is known to be sensitive to microtubule inhibitors (40, 41). The payload consists of DBCO, PEG_4_ linker, Val-Cit dipeptide sequence as cathepsin substrate, p-aminobenzyl alcohol (PAB) self-immolative spacer and the cytotoxic payload MMAE. Site-specific coupling of DBCO-PEG_4_-Val-Cit-PAB-MMAE was accomplished via a chemoenzymatic conjugation approach, resulting in a theoretical drug-to-antibody ratio (DAR) of two (detailed conjugation strategy described in section 'Cytotoxicity of masked and protease-activated CH2-aIgM ADC'). The aIgM ADC should remain masked in systemic circulation, but upon reaching the tumor microenvironment, upregulated protease activity promotes cleavage of the substrate linker and subsequent release of the blocking IgM domain (**Figure 2B**). Following antibody-directed binding to tumor target IgM isotype BCRs, the ADC is expected to be effectively internalized, followed by lysosomal degradation resulting in cleavage of the drug linker and intracellular release of the cytotoxic agent. Finally, MMAE binds to tubulin which inhibits its polymerization and ultimately triggers tumor cell death (42).

### Generation of chicken-derived anti-IgM antibodies

In order to generate protease-activated anti-human IgM antibodies, we screened for IgM binders which are in a second step equipped with the epitope-bearing human IgM domain serving as antigenic affinity-based mask. Antibodies of IgM isotype play important roles in non-immune as well as antigen-induced immune reactions and constant domains of Ig heavy chain are broadly conserved in mammals (43–45). Hence, immunization of popularly chosen mammalian species such as mouse, rabbit or goat might not result in the desired immune response. Accordingly, chickens were considered for immunization as they are phylogenetic distant from humans and previous attempts succeeded in accessing antibodies against conserved epitopes on mammalian molecules (46, 47). Recently, we described the isolation of highly affine antibody fragments derived from immunized chickens using yeast surface display in combination with FACS (48–50). Applying this approach, we obtained high chicken antibody titers against human IgM and were able to enrich binders within two consecutive sorting rounds using 500 nM or 10 nM IgM from human serum, respectively (**Supplementary Figures 1A, B**). Sequence analysis of four yeast single clones emerging from the screening revealed four distinct scFv candidates (S5, S6, S8, S9). The four scFvs were heterologously expressed in *Escherichia coli* and were subjected to B cell binding assays. Antibody clone aIgM S8 was selected as lead candidate since it demonstrated affine binding to IgM^+^ lymphocytes while IgM^-^ cells were not targeted indicating isotypic specificity (**Supplementary Figure 2**).

### Generation and characterization of conditionally activated aIgM

**Figure 3:**
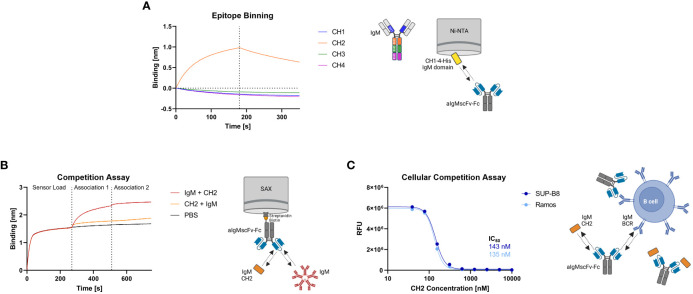
Epitope binning of aIgMscFv-Fc and CH2/IgM competition. **(A)** BLI-assisted epitope binning. The four His-tagged constant IgM domains of the HC (CH1, CH2, CH3 and CH4) were loaded onto Ni-NTA biosensor tips and associated with 150 nM aIgMscFv-Fc, followed by dissociation. **(B)** BLI-assisted competition assay. Biotinylated aIgMscFv-Fc was loaded onto SAX tips and IgM CH2/IgM from human serum were associated in sequence. **(C)** Cellular competition assay. IgM^+^ SUP-B8 and Ramos B cells were incubated with 100 nM aIgMscFv-Fc and varying concentrations of IgM CH2 domain (39-10,000 nM). Detection was performed using anti-human IgG Fc-PE staining and flow cytometry.

The aIgM scFv S8 was reformatted as scFv-Fc fusion and as Fab-Fc full-length antibody. To investigate which of the four constant IgM domains aIgM S8 targets, biolayer interferometry (BLI) epitope binning was performed. To this end, His-tagged CH1-CH4 IgM domains were expressed separately in Expi293F™ cells and cell culture supernatants were immobilized on Ni-NTA biosensors. Association with aIgMscFv-Fc revealed specific and exclusive binding to IgM CH2 domain (**Figure 3A**). Consequently, simultaneous binding of full-length IgM and IgM CH2 domain should not be possible. This was confirmed by loading of biotinylated aIgMscFv-Fc onto SAX biosensors and stepwise association with equimolar concentrations of CH2 in antigens using 1,000 nM single IgM CH2 domain and 100 nM (pentameric) IgM from human serum (**Figure 3B**). The slightly increased binding signal detected when incubating with CH2, following the first IgM association can be ascribed to the small size of IgM CH2 (13 kDa) in comparison to the pentameric IgM molecule (970 kDa) allowing the single Ig domain to bind unoccupied paratopes which are sterically unavailable for pentameric IgM. Attempts to determine the affinity of aIgMscFv-Fc towards IgM CH2 failed as the off-rate turned out to be very low, nevertheless, implying high-affinity binding (**Supplementary Figure 3A**) . In a similar setup, a competition assay with B cells was performed using IgM^+^ SUP-B8 and Ramos cells incubated with 100 nM aIgMscFv-Fc and varying concentrations of IgM CH2 (39-10,000 nM) (**Figure 3C**). In accordance with the BLI measurements, B cell binding decreased with increasing IgM CH2 concentration amounting to IC_50_ values of 143 nM and 135 nM for SUP-B8 and Ramos cells, respectively. Hence, BCRs of IgM isotype on the cell surface compete with the soluble IgM CH2 domain for scFv binding corroborating the notion that CH2 is the epitope-bearing IgM domain.

Taken together, these results indicate that human IgM CH2 domain suits as masking unit for the previously identified aIgM S8 antibody since pre-incubation of antibody with IgM CH2 efficiently impairs IgM binding in biolayer interferometric studies as well as on a cellular level with membrane-bound IgM.

**Figure 4:**
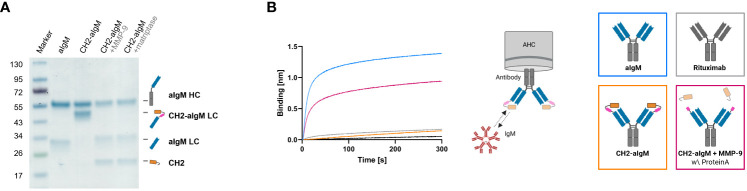
Protease-activation of CH2-masked aIgM. **(A)** Reducing SDS-PAGE of depicted antibodies with schematic representations of heavy and (masked) light chains. **(B)** BLI measurement. The four antibody constructs (Rituximab, aIgM, CH2-aIgM, Protein A purified CH2-aIgM+MMP-9) were loaded onto AHC biosensor tips and associated with 100 nM IgM from human serum.

For masking of aIgM S8 antibody the IgM CH2 domain was fused to the light chain by a linker with dual-protease site. The unmasked aIgM and masked aIgM antibody variant, referred to as CH2-aIgM, were expressed in Expi293F™ cells and purified via Protein A affinity chromatography. Integrity, size and purity of the proteins including stability of the linker during production and purification process were confirmed using reducing SDS-PAGE analysis (**Figure 4A**). Thermal stability investigated by SYPRO Orange revealed melting temperatures of 72.5°C and 71.5°C for the aIgM and CH2-aIgM, respectively (**Supplementary Figure 4**). Thus, no significant change in thermal stability was observed by attachment of the additional Ig domain. The functionality of the parental full-length aIgM concerning binding of IgM from human serum and IgM-derived CH2 domain was confirmed by BLI (**Supplementary Figure 3B**). In order to prove feasibility of reactivation of the aIgM binding capability in the masked antibody, CH2-aIgM was treated with either MMP-9 or matriptase. Linker proteolysis was analyzed by SDS-PAGE demonstrating successful and complete linker cleavage of the CH2-aIgM LC by both proteases which resulted in the aIgM LC migrating slightly higher in SDS gel electrophoresis than the unmasked aIgM LC due to residual linker amino acids, and the solitary CH2 domain (**Figure 4A**). Biolayer interferometry measurements were conducted to investigate, whether the binding capacity of CH2-aIgM is diminished and can in a next step be restored by protease cleavage. Therefore, aIgM, CH2-aIgM, protease treated CH2-aIgM and rituximab as an unrelated control were immobilized onto AHC biosensors and subsequently incubated with IgM from human serum. With CH2-aIgM loaded, association of IgM is completely impaired since the binding signal is comparable to rituximab control (**Figure 4B**). As previous experiments have shown that the dissociation rate of soluble IgM CH2 from the antibody is low, a Protein A purification step was systematically introduced after protease-mediated linker hydrolysis in subsequent assays in order to remove a large fraction of cleaved CH2 domain. MMP-9-cleaved, purified CH2-aIgM allows IgM association, although maximum binding capacity of aIgM may not fully be restored. This effect of reduced interaction might be traced back to remaining cleaved masking units blocking the aIgM paratope due to slow dissociation. Similar results were obtained in BLI experiments associating with different IgM concentrations (3.9-125 nM) for competition with cleaved CH2 masking moiety as well as in a reverse experimental setup immobilizing IgM to the biosensor and incubating with the respective antibody variants (**Supplementary Figures 3C, D**).

### On-cell binding of masked and protease-activated CH2-aIgM

**Figure 5:**
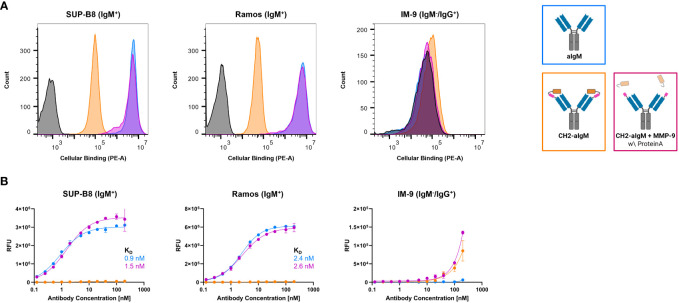
Cellular binding of unmasked and CH2-masked aIgM variants. Flow cytometry analysis of IgM^+^ (SUP-B8, Ramos) and IgM^-^ (IM-9) B cells incubated with aIgM, CH2-aIgM and Protein A purified CH2-aIgM+MMP-9 antibodies and stained via anti-human IgG Fc-PE secondary detection antibody. **(A)** B cells were incubated with 100 nM of respective antibodies. Negative control samples (0 nM, black) represent cells stained with secondary detection antibody only. Histograms were created using FlowJoTM v10 Software (BD Life Sciences). **(B)** Cell titration of respective antibodies (0.125-200 nM) on B cells. On-cell K_D_s were determined using variable slope four-parameter fit. Results are shown as mean RFU, error bars represent standard deviation derived from experimental duplicates. Data is representative of three independent experiments.

To investigate, whether the masked CH2-aIgM remains innate to IgM interaction when membrane-bound in a high copy number on cells and whether protease-activation of CH2-aIgM restores binding functionality, cell binding experiments were performed using flow cytometry. SUP-B8 and Ramos B lymphoid cell lines derived from Burkitt lymphoma were used as IgM^+^ cells while IgM^-^/IgG^+^ IM-9 B cells served as control (51–53). Cells were stained with 100 nM of respective antibody and PE-conjugated secondary antibody for detection. While aIgM represents maximum binding on IgM^+^ SUP-B8 and Ramos cells, the masked variant CH2-aIgM shows 61-fold and 102-fold reduced cell binding, respectively (**Figure 5A**). Upon MMP-9 cleavage and Protein A purification of CH2-aIgM cell binding capacity is fully restored to a maximum binding comparable to the unmasked aIgM version. None of the antibodies showed unspecific interactions with IgM^-^/IgG^+^ IM-9 off-target cells. Furthermore, cell titration was conducted for determination of on-cell affinities for the masked and the proteolytically activated CH2-aIgM. Antibodies were applied to the cells in a serial dilution with concentrations ranging from 0.125 to 200 nM. Apparent binding affinities for aIgM amounted to 0.9 nM for SUP-B8 cells and 2.4 nM for Ramos cells, while titration of cleaved CH2-aIgM resulted in similar values of 1.5 nM and 2.6 nM for SUP-B8 and Ramos, respectively (**Figure 5B**). Besides comparable on-cell K_D_s of aIgM and protease treated CH2-aIgM, maximal binding levels are also restored. The masked CH2-aIgM displayed significantly reduced cell binding indicated by multiple-fold increased on-cell affinity values and decreased saturation binding levels (**Figure 5B**; **Supplementary Figure 5A**). Furthermore, interactions of aIgM and CH2-aIgM with PBMCs isolated from healthy human donor blood were scrutinized revealing binding of aIgM likely to the B cell subpopulation while the blocked aIgM antibody largely spares PBMCs (**Supplementary Figure 5B**). These results suggest that masking the aIgM antibody using a covalently linked blocking domain increases the likelihood of the mask remaining on the antibody due to loss of conformational degrees of freedom and high affinity, and thus significantly reduces binding of IgM. However, the MMP-9 treated CH2-aIgM revealed recovery in binding which indicates dissociation of the linker-cleaved CH2 domain from the antibody by reasons of competition with a high number of IgM BCRs in a cellular context (**Figure 5**). While covalent linkage of the CH2 domain shows efficient masking, presence of the cleaved masking unit reduces cell binding of the unmasked antibody to some extent (**Supplementary Figure 6**). This may be attributed to the relatively high concentration of masked antibody used (100 nM) and the slow dissociation kinetics of the masking CH2 domain.

Overall, transferring the features of the masked IgM antibody in a physiological setting, the blocked antibody is expected to be inert to interactions and interceptions related to IgM in systemic circulation while linker hydrolysis in the tumor microenvironment might result in localized unrestricted binding capacity and robust tumor targeting.

### Cytotoxicity of masked and protease-activated CH2-aIgM ADC

**Figure 6:**
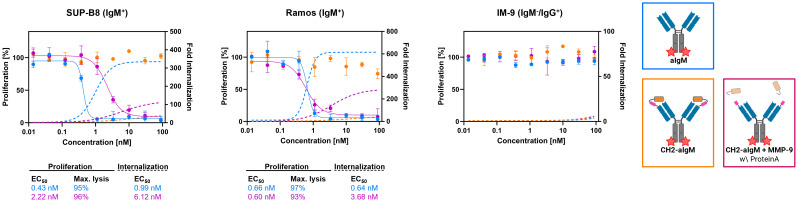
Internalization and cytotoxicity of unmasked and CH2-masked aIgM ADC variants towards B cells. For cytotoxicity studies IgM^+^ (SUP-B8, Ramos) and IgM^-^ (IM-9) B cells were exposed to varying concentrations (0.014-90 nM) of aIgM, CH2-aIgM and Protein A purified CH2-aIgM+MMP-9 MMAE-conjugated antibodies for 72 h. Cell proliferation was normalized to untreated control cells (0 nM). For internalization studies pHAb-conjugated aIgM, CH2-aIgM and Protein A purified CH2-aIgM+MMP-9 (0.014-90 nM) were applied to B cells and incubated overnight. Fold internalization was defined by the ratio of relative fluorescence units (RFU) of the respective antibody sample and the untreated sample without antibody (0 nM). EC_50_s were determined using variable slope four-parameter fit. Results are shown as mean, error bars represent standard deviation derived from experimental duplicates.

For investigation of cytotoxicity mediated by an aIgM ADC and its masked variant CH2-aIgM ADC, both antibody versions were armed with MMAE generating ADCs with an expected DAR of two. Attachment of DBCO-PEG_4_-Val-Cit-PAB-MMAE to the antibodies was accomplished site-specifically by a two-step approach of enzyme-assisted azide modification of the heavy chain's C-terminus which was endowed with a recognition sequence for lipoate-protein ligase A and click chemistry with DBCO-conjugated payload. Prior to cytotoxicity studies aIgM and CH2-masked aIgM were investigated towards internalization properties using our antibodies labeled with pH-dependent dye and flow cytometric analysis (54–56). In IgM^+^ cell lines, the proportion of endocytosed aIgM increased concentration-dependently reaching saturation in the single-digit nanomolar range while significantly less internalization was detected for CH2-aIgM (**Figure 6**). Internalization of aIgM and CH2-aIgM was barely measurable in IgM^-^ B cells. Data points of internalization measurement were removed for clarity but are available in the **Supplementary Material** for all investigated molecules (**Supplementary Figure 7**).

First, *in vitro* cytotoxicity studies were conducted with aIgM-MMAE, its masked variant CH2-aIgM-MMAE as well as a pre-cleaved, Protein A purified CH2-aIgM-MMAE version using on-target SUP-B8 and Ramos cells while IM-9 served as off-target cells. Consistent with the internalization properties of aIgM in target cells, IgM^+^ cells were sensitive to aIgM ADC-induced cell death (**Figure 6**). The aIgM ADC displayed potent dose-dependent cell killing with EC_50_ values amounting to 0.43 nM and 0.66 nM for SUP-B8 and Ramos cells, respectively. No significant reduction in cell proliferation was observed by application of the aIgM-MMAE molecule to IM-9 B cells not expressing IgM. Paratope-masked aIgM ADC was unable to mediate cell death in any cell line, which we expected since no endogenous proteolytic activity was observed in cell culture supernatants supplemented with CH2-aIgM during 72 h of incubation (data not shown). Notably, MMP-9 and matriptase activity was detected in B cell lymphoma tumor tissue warranting the concept of protease-mediated antibody activation (33, 34). The activity of aIgM was mostly restored after linker hydrolysis since CH2-aIgM pre-treated with MMP-9 resulted in significantly decreased survival of IgM^+^ cells. Comparing potencies of the parental unmasked ADC to the pre-cleaved CH2-aIgM, an approximately 5-fold reduced cytotoxic effect was observed on SUP-B8 cells, whilst on Ramos cells efficacy was fully recovered (**Figure 6**). Besides comparable induction of lymphoma cell killing in EC_50_ values, similar levels in maximal cell lysis were observed. MMP-9 treated unpurified CH2-aIgM ADC, revealed 8-9-fold increased half maximal effective doses compared to the parental unmasked ADC in target lymphoma cells (**Supplementary Figure 8**).

**Figure 7:**
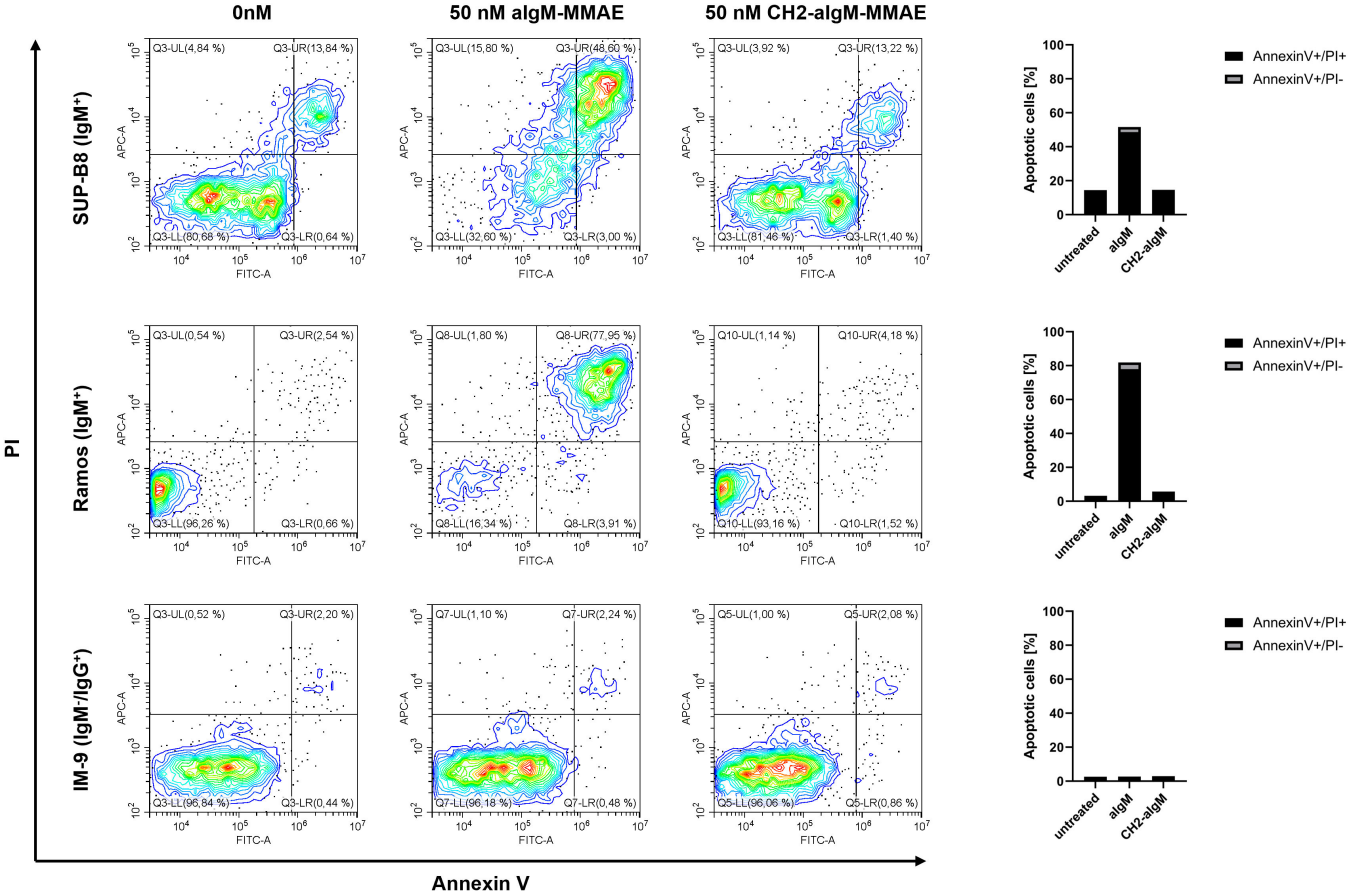
Apoptosis induction of aIgM and CH2-masked aIgM ADC in B cells. IgM^+^ (SUP-B8, Ramos) and IgM^-^ (IM-9) B cells were exposed to 0 nM, 50 nM of aIgM-MMAE and 50 nM CH2-aIgM-MMAE for 72 h. Cells were stained with Annexin V-FITC and propidium iodide (PI) and analyzed by flow cytometry. Percentage of Annexin V-FITC+/PI+ and Annexin V-FITC+/PI- (apoptotic cells) is depicted in right bar chart. Data is representative of two independent experiments.

Next, we investigated whether apoptosis was triggered by aIgM-MMAE and CH2-aIgM-MMAE. To this end, cells expressing BCRs of IgM and IgG isotype were treated with the respective ADCs for 72 h and analyzed by Annexin V-FITC and propidium iodide (PI) staining using flow cytometry. Application of 50 nM aIgM-MMAE resulted in increased fractions of Annexin V-FITC-positive IgM^+^ cells, indicating that apoptosis was induced by antibody-guided chemotherapeutic damage (**Figure 7**). SUP-B8 and Ramos cells being exposed to aIgM-MMAE showed approximately 4-fold and 26-fold increase in Annexin V-FITC positivity, respectively, compared to untreated control cells (0 nM). Previous investigations have postulated that MMAE induces cell death through a rarely studied mechanism termed mitotic catastrophe possibly being a prelude mechanism to apoptotic or necrotic cell death and further includes signs of autophagy (57–60). In contrast, CH2 masked aIgM ADC did not induce any killing detectable by Annexin V-FITC or PI staining. Likewise, IM-9 IgM^-^/IgG^+^ off-target cells remained unaffected during aIgM ADC treatment.

## Discussion

Overcoming the limitations of treatment paradigms for B cell NHL, novel approaches of highly potent immunotherapies that work in concert with the host immune system such as bispecific T cell engaging antibodies and chimeric antigen receptor (CAR) T cells have been introduced (61–64). Great efforts have further been made in probing antibody-drug conjugates for lymphoma therapies. Brentuximab vedotin, Polatozumab vedotin and Loncastuximab tesirine represent FDA-approved ADCs to treat different types of B cell lymphoma, targeting antigens such as CD30, which is expressed by activated B cells, CD79b, and CD19, present on all B cell types apart from pre-proB cells and mature plasma cells (32, 65–67).

Besides selection of an appropriate antibody ensuring to reach the tumor target site without affecting healthy cells in the periphery, linker and cytotoxic payload are key design parameters in ADCs. Related to those criterions is the DAR which plays a pivotal role determining ADC's potency, safety, and pharmacokinetics. In general, higher drug loading comes along with increased anti-tumor activity. However, improvement in efficacy is limited and excessive cytotoxic payload may cause instabilities and aggregation and further lead to inferior pharmacokinetics such as in plasma clearance and tumor exposure (68, 69). *Bryant et al.* demonstrated that a DAR of 4 in a (trastuzumab) conjugate revealed highest potency *in vitro* and a significantly increased *in vivo* efficacy compared to the lower DAR conjugates (70). Referred to as the first approved mAb for cancer patients, auristatin-based rituximab ADCs have been developed with DARs of 7-7.5 and 4.2, respectively, both demonstrating potent therapeutic efficacy *in vitro* and *in vivo* (60, 71). Hence, further improvements may be reached for the CH2-masked aIgM ADC by examination of optimal drug loading but were out-of-scope for this proof-of-concept study.

To further promote safety and efficacy of ADCs several innovative approaches have been developed in the last decades. One of them includes the generation of a bispecific ADC that targets HER2 as tumor-associated antigen and CD63 rendering enhanced lysosomal delivery (72). Another appealing tool is introduced by CytomX Therapeutics with the probody platform expanding the availability of new targets for ADCs by antibody paratope masking and tumor-specific protease-activation. Probody-drug conjugates are supplied with a protease cleavable linker connected to a peptide mask limiting target engagement in normal tissue and circulation (73). CX-2029 targeting transferrin receptor 1 (CD71) attached to MMAE is currently being investigated in phase II clinical trials displaying translational and clinical activity at tolerable doses in patients (27, 73).

In this study, we present a novel conditionally activated anti-IgM antibody-drug conjugate for precise B cell lymphoma elimination. To this end, we isolated a chicken-derived IgM-specific antibody (aIgM), which was further fused to the epitope-holding IgM domain CH2 by a tumor-protease cleavable linker ultimately equipped with the cytotoxic payload MMAE. Efficient blockage of the tumor targeting moiety in CH2-aIgM was confirmed by biolayer interferometry. The masked antibody regained activity upon protease treatment, displaying affine binding to IgM from human serum. On a cellular level CH2-aIgM was inert to interact with IgM^+^ B cells while the cleaved variant revealed excellent on-cell affinities comparable to the parental unmasked antibody regarding on-cell affinity constants in the low single-digit nanomolar range as well as maximum binding capacities. This allows penetration into the tumor microenvironment without being captured by soluble IgM or non-malignant IgM^+^ B cells ultimately improving pharmacokinetic properties. Reaching the tumor target site, tumor-protease-mediated linker hydrolysis engenders high affinity targeting. The aIgM ADC demonstrated specific and effective receptor-mediated cellular uptake which was closely linked to killing of lymphoma cells exhibiting strong signs of apoptotic cell death. Cytotoxicity of the inactive ADC version was shown to be reduced since no cell killing was observed in the investigated concentration range, thus potentially preventing systemic side effects. CH2-aIgM is rendered active by proteases leading to regained toxicity towards malignant IgM^+^ B lymphocytes. Further animal studies are required to reveal whether the restrictive and potent *in vitro* anti-tumor efficacy of the antibody introduced in this study can be confirmed *in vivo*.

Our results further show that it is feasible to generate proteolytically activated antibody-drug conjugates against immunoglobulins of isotype (Ig)M for B cell lymphoma treatment. This novel strategy of Ig targeting in B cell-derived malignancies may be superior to conventional approaches in several respects. By addressing only a fraction of B cells, unwanted on-target off-tumor effects are reduced which is further enhanced through the masking functionality while conventional pan-B cell targeting results in patients suffering from B cell-aplasia induced immunosuppression (74). In case of the anti-CD20 antibody rituximab, various resistance mechanisms are existing such as tumor-dependent alterations e.g., antigen downregulation and antigenic modulation or host-dependent immunologic factors e.g., Fc receptor polymorphisms (75–79). Alternative attempts addressing the BCR include patient-specific anti-idiotypic peptides or antibodies against variable regions, however, laborious and time-consuming manufacturing may limit developability (80–83). We propose an alternative mechanism of tumor clearance providing the possibility to therapy relapsed or refractory NHL in the second- or third-line setting solely implying BCR sequencing to identify the disease-causing B cell clone. This concept would be effortlessly applicable to different kinds of B cell-derived malignancies as there are only four human Ig isotypes (IgM, IgG, IgD, IgA) expressed as BCRs, against which antibodies are already available and can in a next step be masked by the respective epitope-bearing Ig domains. As for the CH2-masked aIgM antibody, further protein or antibody engineering may be required to fine-tune the affinity, particularly concerning the off-rate of the blocking moiety to the antibody. In this *in vitro* study, the cleaved IgM CH2 masking unit not being removed from the assay sample associates to the aIgM paratope and thus hampers full functionality of the antibody in terms of (cell) binding and cytotoxicity requiring further purification to decrease the molar ratio of CH2 to corresponding aIgM antibody. In the body, demasking is mediated by proteases such as MMP-9 and matriptase described to be prognostic factors for B cell lymphoma when overexpressed (33, 34). The mechanisms of masking domain release may be shaped by multiple variables *in vivo*. Each individual binding event is a one-step reversible biomolecular process obeying the law of mass action. While interaction to cut CH2 is of monovalent nature, binding to IgM on B cells involves both antibody valences showing avidity effects. Moreover, unrestricted diffusion of the soluble masking domain in blood is opposed to spatial clustered B cell surface receptors effecting rebinding of aIgM which likely leads to local dilution of the masking domain ultimately resulting in preferred cell binding (84, 85). Contrary to synthetic peptide masks, the Ig domain used for paratope-blocking is of human origin reducing the risk of immunogenicity. However, aIgM is a chimeric antibody constituted of chicken-derived variable domains fused to human IgG1 constant domains. Hence, humanization is required to minimize immunogenicity in therapeutic applications. Our group recently developed a straightforward method to humanize avian-derived antibodies by CDR grafting onto a human germline framework based on Vernier residue randomization that could be applied for this purpose but is beyond the scope of this study (86, 87).

Taken together, our approach demonstrates a novel mechanism to specifically eradicate NHL B cells while preserving healthy human B lymphocytes that do not display IgM isotype BCRs. Constituting an inactive anti-IgM antibody-drug conjugate which is actuated in the proteolytic tumor environment, the molecule unites an enhanced safety profile due to tumor-proximity restricted activation and potent anti-tumor efficacy relying on a highly cytotoxic payload. Furthermore, our study provides a basis for the development of protease-activated anti-Ig ADCs for the treatment of B cell-driven pathologies.

## Materials and methods

### Chicken immunization and yeast library construction

Chicken immunization and scFv yeast surface display library generation were performed as described previously (48). In brief, an adult chicken (*Gallus gallus domesticus*) was immunized with IgM from human serum (Sigma Aldrich) on days 1, 14, 28, 35, and 56. The animal was sacrificed on day 63, followed by isolation of the spleen and total RNA extraction. The immunization process as well as splenic RNA isolation were executed by Davids Biotechnologie GmbH (Regensburg, Germany). For library construction, RNA was reverse transcribed to cDNA. Subsequently, genes encoding VH and VL were amplified and transferred into a YSD vector (pCT) via homologous recombination in yeast (*Saccharomyces cerevisiae* strain EBY100). Library generation in EBY100 cells was conducted according to Benatuil and colleagues (88). Cultivation and general handling of yeast cells are described elsewhere (48, 83).

### Yeast library screening

Induction of gene expression and scFv surface presentation was achieved by inoculation of yeast cells in Synthetic Galactose minimal medium with Casein Amino Acids (SG-CAA) at an OD_600_ of 1.0 and incubation overnight at 30°C and 180 rpm. For library sorting, cells were harvested by centrifugation and washed with PBS+0.1% (w/v) BSA (PBS-B). Antigen staining was conducted with DyLight650™-labelled IgM from human serum (Sigma Aldrich) conjugated beforehand using 5-fold excess of DyLight650™ NHS Ester (Thermo Fisher Scientific). Simultaneously, staining for surface presentation using anti-cMyc antibody FITC-conjugated (Miltenyi Biotec; diluted 1:50) was performed for 30 min on ice. After another PBS-B washing step, the yeast library was screened using BD Influx cell sorter with corresponding BD FACS Sortware v1.0.

### Expression and purification of scFv, scFv-Fc and Fab-Fc variants

Reformatting, expression and purification of scFvs was performed as described previously (89). Briefly, isolated yeast vectors were sequenced and scFv encoding genes were reformatted into a pET30 plasmid using golden gate assembly, followed by recombinant expression in *E. coli* SHuffle^®^ T7 Express (New England Biolabs). A two-step affinity purification was performed including IMAC and Strep-Tactin^®^XT purification, followed by buffer exchange against PBS. Production of Fc-fused scFvs and full-length antibodies (Fab-Fc) was conducted with pTT5-derived golden gate assembly vectors in Expi293F™ cells (Thermo Fisher Scientific). Expi293F™ cells were transiently transfected using ExpiFectamine™ 293 Transfection Kit (Thermo Fisher Scientific) following the manufacturer's protocol. For purification of Fc-containing antibody constructs, cell culture supernatants were collected five days post transfection, sterile filtered and applied to a HiTrap™ Protein A HP column (GE Healthcare) using an ÄKTA pure™ chromatography system (GE Healthcare). Buffer exchange against PBS or TBS was performed using a HiTrap™ Desalting column (GE Healthcare).

### Cell lines

B cells including SUP-B8, IM-9 and Ramos cells were cultured at 37°C and 5% CO_2_. All B cell lines were maintained in RPMI-1640 supplemented with 15% FBS and 1% Penicillin-Streptomycin and sub-cultured every 2-3 days. Expi293F™ cells were cultured in Expi293™ Expression Medium (Thermo Fisher Scientific), sub-cultured every 3-4 days and incubated at 37°C and 8% CO_2_.

### Protease-mediated protein hydrolysis

Recombinant human MMP-9 (Acro Biosystems) or recombinant human matriptase/ST14 catalytic domain (Bio-Techne) were used to cleave the dual-protease cleavable linker of CH2-aIgM. Prior to the protein hydrolysis reaction, MMP-9 was pre-activated with 1 mM 4-aminophenylmercuric acetate (APMA) overnight at 37°C. Proteins were dissolved in TBS pH 7.4, if necessary, by buffer exchange, ensuring suitable conditions for the MMP-9 and matriptase hydrolysis reaction. 0.25 mg of the respective antibody variant was mixed with 0.25 μg (0.1 mg/ml) of activated human MMP-9 or matriptase. Protein cleavage was performed at 37°C for 48 h. Complete linker hydrolysis was confirmed using SDS-PAGE under reducing conditions. Cleaved CH2-aIgM protein was further purified using Protein A spin columns (Protein A HP SpinTrap, Cytiva) in order to remove fractions of the masking IgM CH2 domain.

### Thermal shift assay

Experiments to determine thermal stability were performed using a CFX Connect Real-Time PCR Detection System (BioRad) with a temperature gradient from 20°C to 95°C and 0.5°C/10 s. The derivatives of the melt curves were calculated with the corresponding BioRad CFX Maestro software to determine the melt temperature (Tm). All reactions were performed in PBS in presence of 0.1 mg/ml protein and SYPRO Orange (Thermo Fisher Scientific, diluted 1:100).

### Biolayer interferometry

For biolayer interferometric measurements the Octet RED96 system (ForteBio, Sartorius) was used. Therefore, respective biosensor tips were soaked in PBS pH 7.4 for at least 10 min before assay start.

For epitope binning, Ni-NTA Biosensors (NTA, Sartorius) were loaded with cell culture supernatants of single His-tagged IgM domains expressed in Expi293F™ cells. All following steps were performed using kinetics buffer (KB, Sartorius). Association was measured for 180 s with 150 nM aIgMscFv-Fc followed by dissociation for 180 s.

For the CH2/IgM competition assay, High Precision Streptavidin biosensors (SAX, Sartorius) were loaded biotinylated aIgMscFv-Fc. After quenching in KB, two association steps of 250 s were conducted in sequence, a first association step using either 100 nM IgM from human serum (Sigma Aldrich) or 1,000 nM IgM CH2 was followed by a second association using 1,000 nM IgM CH2 or 100 nM serum, respectively.

For affinity determination of aIgMscFv-Fc and aIgMFab-Fc anti-human IgG Fc capture biosensors (AHC, Sartorius) were used to immobilize the aIgM antibodies. After a quenching step in KB, an association step using CH2-His with concentrations ranging from 31.25 to 500 nM or IgM from human serum (Sigma Aldrich) was performed followed by a dissociation step in KB. Association in KB served as reference and was subtracted prior to evaluation steps. Data analysis was performed using ForteBio data analysis software 9.0. Binding kinetics including the equilibrium constant K_D_ were determined using Savitzky-Golay filtering and 1:1 Langmuir model.

To confirm that the parental full-length aIgM antibody binds to IgM and IgM-derived CH2, aIgM antibody was loaded onto AHC biosensor tips, followed by quenching in KB, association with 50 nM IgM from human serum or 250 nM IgM CH2 and dissociation in PBS. In the same experimental setup, binding of aIgM, CH2-aIgM, non-purified and Protein A purified CH2-aIgM+MMP-9 and rituximab (control) were evaluated for IgM binding by association of 100 nM or 3.9-125 nM IgM from human serum. In a reverse experimental setup, biotinylated IgM from human serum was loaded onto SAX biosensor tips. After a quenching step in KB, 100 nM of the respective antibody variants were associated.

### PBMC isolation

Peripheral blood mononuclear cells (PBMCs) were isolated from buffy coats from healthy human donors supplied by the Deutsche Rotes Kreuz (Frankfurt). To this end, 25 ml blood was mixed 1:1 with PBS+2% (w/v) FBS and PBMCs were purified using SepMate-50 tubes following the manufacturer's instructions (StemCell Technologies).

### Cellular binding

Cellular binding of the antibodies was determined by affinity titration using IgM^+^ SUP-B8 and Ramos cells. IgM^-^ (IgG^+^) IM-9 cells were used to analyze unspecific cell binding. To this end, cells (1.5x10^5^ cells/well) were washed with PBS-B and subsequently incubated with the respective antibody constructs in varying concentrations (for cell titration: 0.125-200 nM, serial dilution) for 30 min on ice. Followed by another PBS-B washing step, anti-human IgG Fc PE-conjugated secondary antibody (Thermo Fisher Scientific, diluted 1:50), anti-his AF647-conjugated secondary antibody (Thermo Fisher Scientific, diluted 1:50) or Streptavidin-APC conjugate (Thermo Fisher Scientific, diluted 1:50) was applied for 20 min on ice. After final washing with PBS-B, flow cytometry was performed using CytoFLEX S System (Beckman Coulter). The relative fluorescence units (RFU) were plotted against the respective logarithmic antibody concentration. The resulting curves were fitted with a variable slope four-parameter fit using GraphPad Prism.

### Internalization assays

Investigations towards receptor-mediated antibody internalization were performed using pHAb Amine Reactive dye (Promega) according to the manufacturer's instructions. In brief, aIgM, CH2-aIgM, non-purified and Protein A purified CH2-aIgM+MMP-9 were conjugated with pHAb dyes and applied to B cells (2x10^4^ cells/well) in different concentrations (0.014-90 nM) in a 96-well plate. After incubation overnight, cells were washed once with PBS and internalization was measured using flow cytometry. Fold internalization was determined by the ratio of relative fluorescence units (RFU) of the respective antibody sample and the untreated sample without antibody (0 nM). The resulting curves were fitted with a variable slope four-parameter fit and EC_50_s were calculated using GraphPad Prism.

### Generation of antibody-drug conjugates

Antibody-drug conjugates were generated via a two-step approach of enzymatic modification and click chemistry for conjugation of monomethyl auristatin E (MMAE) to the Fc fragment. Therefore, the C-terminus of the antibody heavy chain was genetically fused with a lipoic acid ligase acceptor peptide (LAP) serving as recognition sequence for lipoate-protein ligase A (LplA) from *Escherichia coli* (90). Lipoic acid ligase reaction was conducted with 0.1 equivalents (eq.) of a mutant lipoic acid ligase A (LplA^W37V^) (91) accepting various carboxylic acid derivatives in the presence of 5 mM ATP, 5 mM Mg(Ac)_2_ and 10-20 eq. azide-bearing lipoic acid derivative (synthesized in-house) in PBS pH 7.4 for 1h at 37°C. Covalent protein azide-functionalization was confirmed by hydrophobic interaction chromatography followed by click reaction with 5 eq. DBCO-PEG_4_-Val-Cit-PAB-MMAE on Protein A resin (Protein A HP SpinTrap, Cytiva) overnight at 4°C. After acidic elution of ADC from Protein A column the buffer was exchanged to PBS pH 7.4.

### Cytotoxicity assays

Cytotoxic effects of aIgM ADCs were evaluated by exposing IgM^+^ lymphoma B cells or off-target (IgM^-^) cells to different ADC concentrations. Cell viability was analyzed 72 h post ADC addition by a colorimetric method using CellTiter 96^®^ AQ_ueous_ One Solution Cell Proliferation Assay (Promega). Briefly, cells were seeded (1x10^4^ cells/well) in a 96-well plate with the desired antibody concentrations ranging from 0.014-90 nM in a serial dilution. After 72 h, MTS solution was added to the cells and plate was incubated for 2 h. Absorption was measured at 490 nm using CLARIOstar plus microplate reader (BMG LABTECH). Cell proliferation was normalized to untreated control cell absorption values. The resulting curves were fitted with a variable slope four-parameter fit and EC_50_s were calculated using GraphPad Prism.

### Apoptosis assays

For AnnexinV-FITC/PI staining ROTITEST^®^ Annexin V (Carl Roth GmbH + Co. KG) was applied for apoptosis detection of B cells according to the manufacturer's instructions. The analysis was performed using CytoFLEX S System (Beckman Coulter).

## Data availability statement

The raw data supporting the conclusions of this article will be made available by the authors, without undue reservation.

## Ethics statement

Ethical approval was not required for the studies on animals because animal (chicken) immunization was performed by Davids Biotechnologie GmbH. Experimental procedures and animal care were in accordance with EU animal welfare protection laws and regulations. 

## Author contributions

KS: Conceptualization, Investigation, Data curation, Writing - original draft. JuH: Investigation, Writing - review & editing. JaH: Investigation, Writing - review & editing. AE: Conceptualization, Writing - review & editing. HK: Conceptualization, Project administration, Writing - original draft.
