# Supplementary material for: Conditional activation of an anti-IgM antibody-drug conjugate for precise B cell lymphoma targeting
Source: Front Immunol. 2023 Sep 28;14:1258700. doi: 10.3389/fimmu.2023.1258700 (PMC10569071; doi:10.3389/fimmu.2023.1258700)
Supplement: Supplementary file 1 [file DataSheet_1.pdf]

Supplementary Material

Conditional activation of an anti-IgM antibody-drug conjugate for precise B cell lymphoma targeting

Katrin Schoenfeld, Julia Harwardt, Jan Habermann, Adrian Elter and Harald Kolmar*

***Correspondence:** Harald Kolmar: harald.kolmar@tu-darmstadt.de

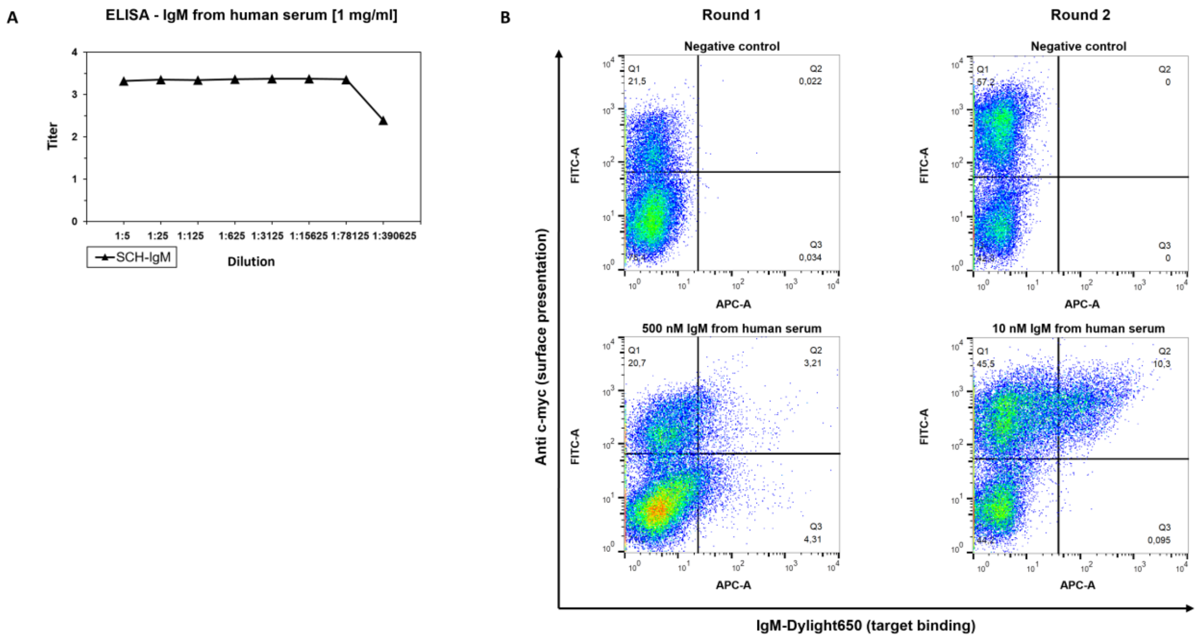

Supplementary Figure 1. Chicken immune response and screening of IgM immune library. (A) Enzyme-linked immunosorbent assay (ELISA) for determination of antibody titer in serum of immunized chickens against IgM from human serum. Experiment was conducted by Davids Biotechnologie GmbH. (B) Sorting of the chicken-derived yeast surface-displayed scFv library. Surface presentation was analyzed using anti c-myc FITC-conjugated antibodies and antigen binding was detected by directly Dylight650-labelled IgM from human serum. Dot plots were created using FlowJoTM v10 Software (BD Life Sciences).

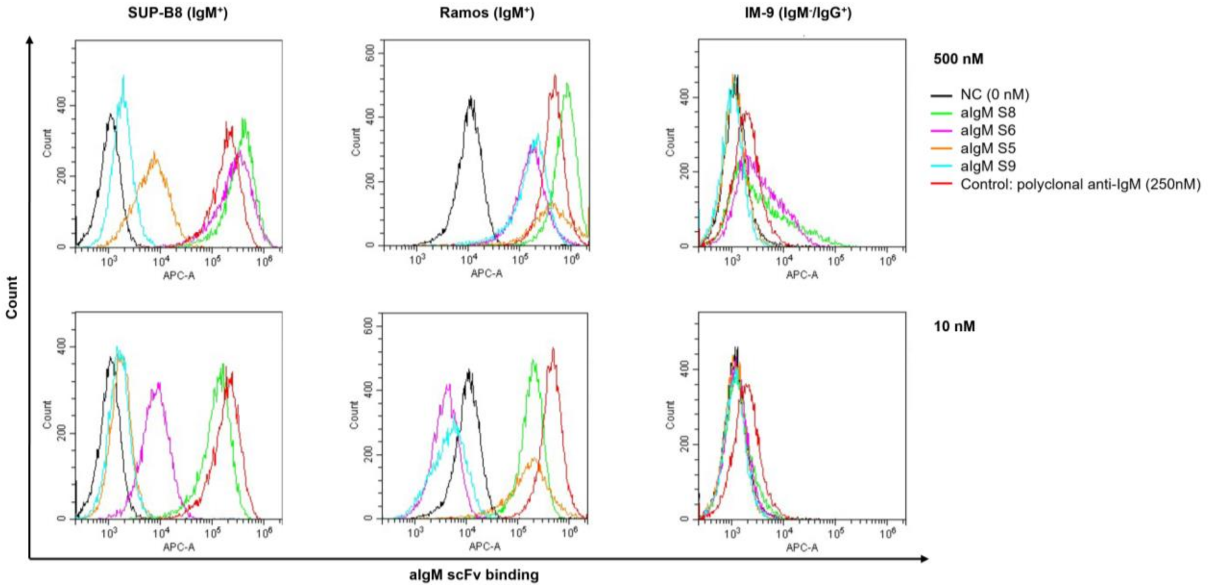

Supplementary Figure 2. Cellular binding of aIgM scFv candidates. Flow cytometry analysis of IgM^+ (SUP-B8, Ramos) and IgM^- (IM-9) B cells incubated with 500 nM and 10 nM of aIgM scFv candidates S8, S6, S5, S9 as well as with 250 nM polyclonal anti-IgM antibody serving as positive control. Negative control samples (0nM, black) represent cells stained with secondary detection antibody only. Staining was conducted via anti-his AF647-conjugated secondary antibody.

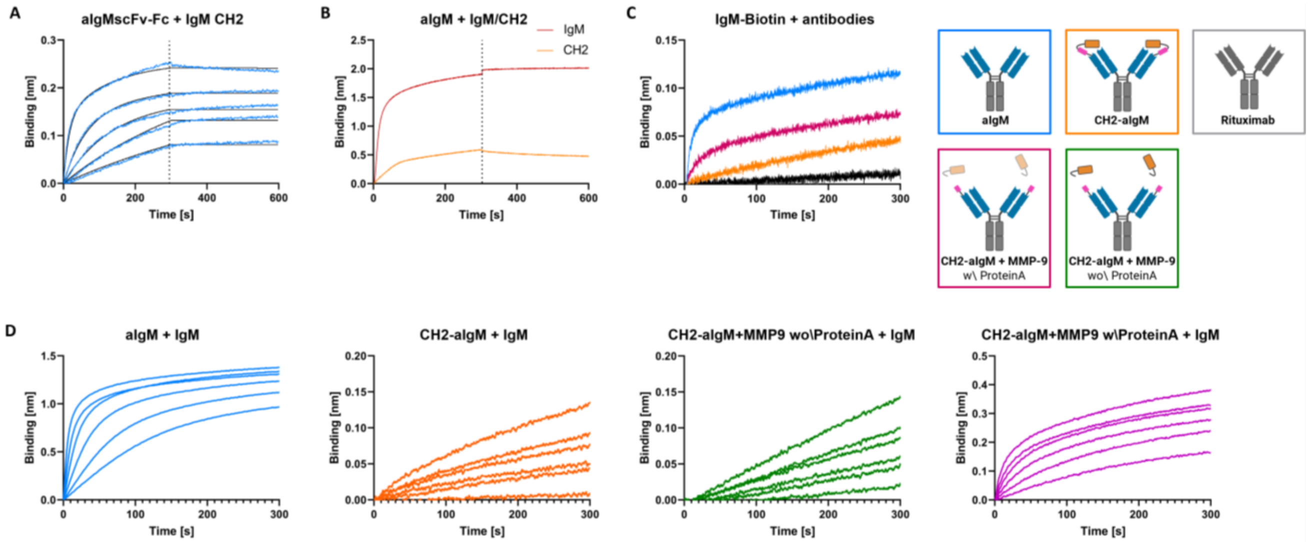

Supplementary Figure 3. Characterization of antigen binding of the aIgM antibody by BLI measurements. (A) Binding kinetics. aIgMscFv-Fc was loaded onto AHC biosensor tips and associated with 31.25 to 500 nM IgM CH2 domain, followed by dissociation in KB. (B) BLI measurement of parental full-length aIgMFab-Fc. aIgM was immobilized to AHC biosensors and associated with 50 nM IgM from human serum or 250 nM IgM CH2, followed by dissociation in PBS. (C) BLI measurement in reverse experimental setup. Biotinylated IgM from human serum was loaded onto SAX biosensor tips and associated with 100 nM of the four antibody constructs (Rituximab, aIgM, CH2-aIgM, Protein A purified CH2-aIgM+MMP-9). (D) BLI measurement using different IgM concentrations. The four antibody constructs (aIgM, CH2-aIgM, non-purified and Protein A purified CH2-aIgM+MMP-9) were loaded onto AHC biosensor tips and associated with 3.9 nM - 125 nM IgM from human serum.

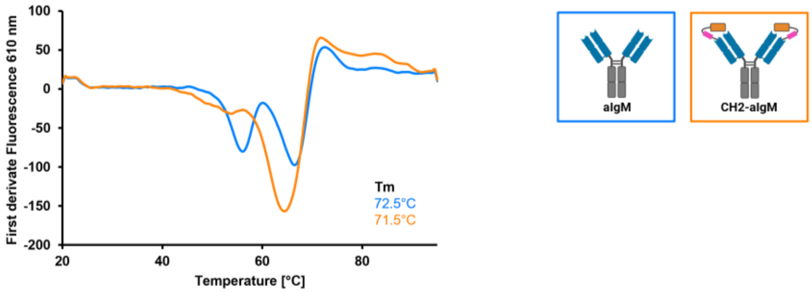

Supplementary Figure 4. Thermal shift assay. Thermal stability was analyzed by means of a temperature gradient from 20°C to 95°C and 0.5°C/10 s. Derivatives of melt curves and melting temperatures (Tm) were determined using the BioRad CFX Maestro software.

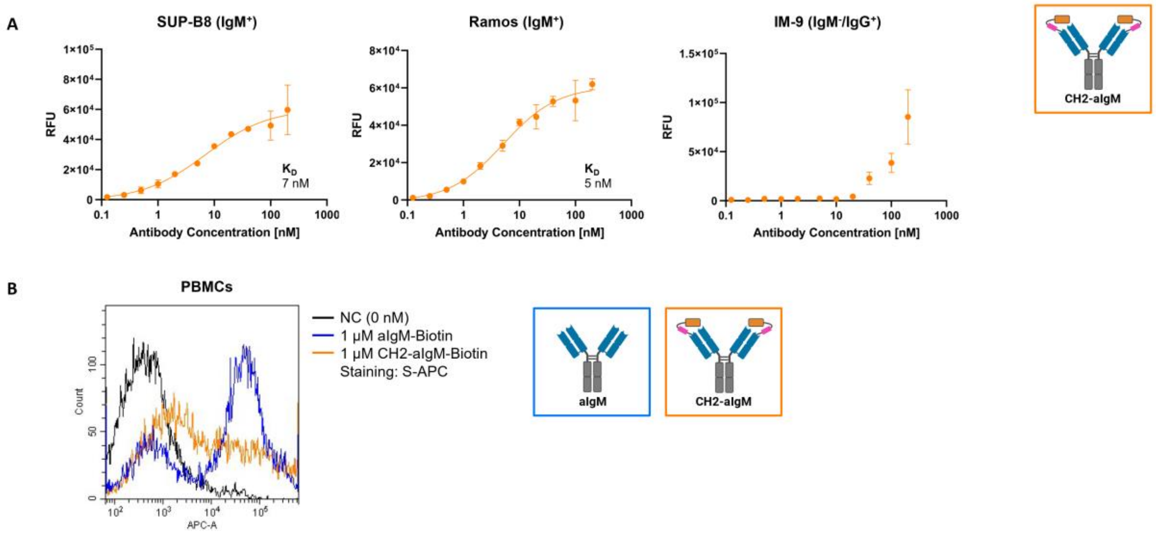

Supplementary Figure 5. Cellular binding of CH2-masked aIgM. (A) Flow cytometry analysis by cell titration of 0.125-200 nM CH2-aIgM on IgM+ (SUP-B8, Ramos) and IgM- (IM-9) B cells. Staining was conducted via anti-human IgG Fc-PE secondary detection antibody. On-cell K_Ds were determined using variable slope four-parameter fit. Results are shown as mean RFU, error bars represent standard deviation derived from experimental duplicates. (B) Flow cytometry analysis of PBMCs incubated with biotinylated aIgM and CH2-aIgM (1µM) and stained by Streptavidin-APC. Negative control samples (0nM, black) represent cells stained with secondary detection reagent only.

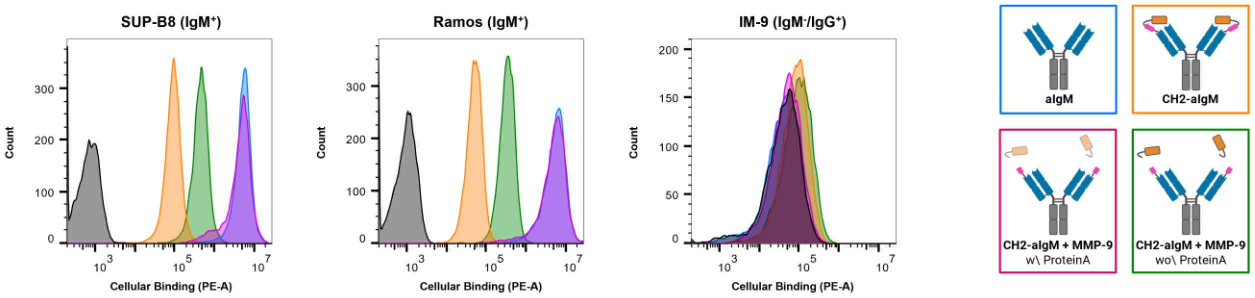

Supplementary Figure 6. Cellular binding of unmasked and CH2-masked aIgM variants to B cells. Flow cytometry analysis of IgM+ (SUP-B8, Ramos) and IgM- (IM-9) B cells incubated with aIgM, CH2-aIgM, Protein A purified and non-purified CH2-aIgM+MMP-9 antibodies and stained via anti-human IgG Fc-PE secondary detection antibody. B cells were incubated with 100 nM of respective antibodies. Negative control samples (0nM, black) represent cells stained with secondary detection antibody only.

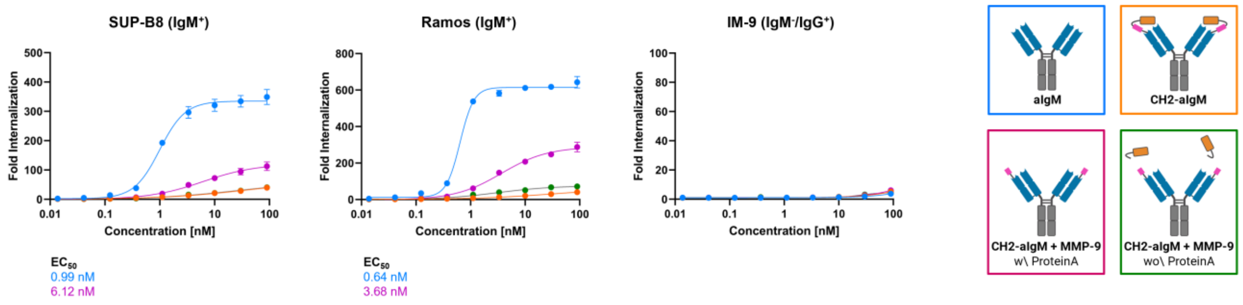

Supplementary Figure 7. Internalization assays of unmasked and CH2-masked aIgM antibody variants in B cells. Internalization of pHab-conjugated aIgM, CH2-aIgM, Protein A purified and non-purified CH2-aIgM+MMP-9 (0.014-90 nM) via endocytosis was analyzed overnight using IgM^{+} (SUP-B8, Ramos) and IgM^{-} (IM-9) B cells. Fluorescence of pH-sensitive dye indicating internalization was measured by flow cytometry. Fold internalization was defined by the ratio of relative fluorescence units (RFU) of the respective antibody sample and the untreated sample without antibody (0 nM). EC_{50}s were determined using variable slope four-parameter fit. Results are shown as mean, error bars represent standard deviation derived from experimental duplicates.

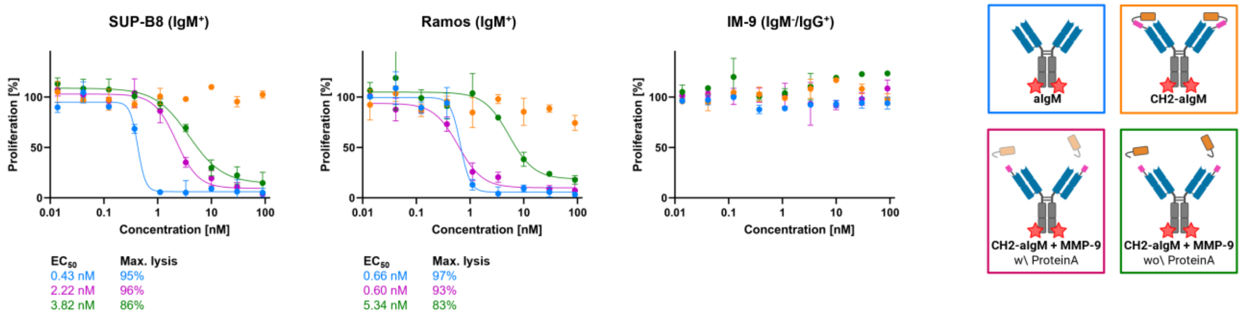

Supplementary Figure 8. Cytotoxicity of unmasked and CH2-masked aIgM ADC variants towards B cells. IgM^{+} (SUP-B8, Ramos) and IgM^{-} (IM-9) B cells were exposed to varying concentrations (0.14-90 nM) of aIgM, CH2-aIgM, Protein A purified and non-purified CH2-aIgM+MMP-9 MMAE-conjugated antibodies for 72 h. EC_{50}s were determined using variable slope four-parameter fit. Results are shown as mean, error bars represent standard deviation derived from experimental duplicates.
